# Plasmonic Hybrids of MoS_2_ and 10-nm Nanogap Arrays for Photoluminescence Enhancement

**DOI:** 10.3390/mi11121109

**Published:** 2020-12-15

**Authors:** Yang Yang, Ruhao Pan, Shibing Tian, Changzhi Gu, Junjie Li

**Affiliations:** 1 Beijing National Laboratory for Condensed Matter Physics, Institute of Physics, Chinese Academy of Sciences, P.O. Box 603, Beijing 100190, China; yang.yang@iphy.ac.cn (Y.Y.); panruhao@iphy.ac.cn (R.P.); tianshibing@iphy.ac.cn (S.T.); czgu@iphy.ac.cn (C.G.); 2 School of Physical Sciences, University of Chinese Academy of Sciences, Beijing 100049, China; 3 Songshan Lake Materials Laboratory, Dongguan 523808, China

**Keywords:** monolayer MoS_2_, 10-nm nanogap, localized surface plasmon resonance, photoluminescence

## Abstract

Monolayer MoS_2_ has attracted tremendous interest, in recent years, due to its novel physical properties and applications in optoelectronic and photonic devices. However, the nature of the atomic-thin thickness of monolayer MoS_2_ limits its optical absorption and emission, thereby hindering its optoelectronic applications. Hybridizing MoS_2_ by plasmonic nanostructures is a critical route to enhance its photoluminescence. In this work, the hybrid nanostructure has been proposed by transferring the monolayer MoS_2_ onto the surface of 10-nm-wide gold nanogap arrays fabricated using the shadow deposition method. By taking advantage of the localized surface plasmon resonance arising in the nanogaps, a photoluminescence enhancement of ~20-fold was achieved through adjusting the length of nanogaps. Our results demonstrate the feasibility of a giant photoluminescence enhancement for this hybrid of MoS_2_/10-nm nanogap arrays, promising its further applications in photodetectors, sensors, and emitters.

## 1. Introduction

In the past ten years, two-dimensional (2D) transition metal dichalcogenides (TMDs) have received plenty of research interest, due to their striking physical properties and applications in optoelectronic devices [1,2]. Molybdenum disulphide (MoS_2_) is a representative member of the TMDs family [3,4], in which the bandgap can transit from indirect to direct [5,6], when the thickness is reduced to a monolayer. The bandgap shifts from 1.29 eV for the bulk MoS_2_ to 1.9 eV for the monolayer MoS_2_, accompanied with an enhancement of the photoluminescence (PL) up to 10^4^ [5]. Therefore, the direct-bandgap characteristic of the monolayer MoS_2_ leads to attractive applications in phototransistors [7], photodetectors [8], light emitters [9], and photocatalysis [10]. However, the thickness of 2D MoS_2_ is too thin to absorb sufficient light, which limits the light-harvest efficiency and consequently restricts its practical applications. Therefore, efficiently enhancing the light absorption and photoluminescence (PL) emission of MoS_2_ has become an important issue for exploring the practical applications in optoelectronic devices. In recent years, integrating MoS_2_ with plasmonic nanoscale metals has been demonstrated to be an effective route to promote the optical properties of MoS_2_ [11,12,13].

Plasmonic nanoscale metals, including noble metal nanoparticles and nanostructures, can strongly enhance the electromagnetic (EM) fields of the excitation light, due to the localized surface plasmon resonance (LSPR) on the surface of nanoscale metals [14,15,16]. Gao et al. have prepared the hybrids of MoS_2_ and Ag nanoparticles, including shape-controlled cubes, octahedra, and spherical particles, and systematically studied the influences of the morphology of nanoparticles on the PL emission of MoS_2_ [17]. Compared with nanoparticles, nanostructures fabricated using advanced nanofabrication techniques exhibit higher controllability, reproducibility, and large-scale periodicity [18]. By modulating the diameter of nanodiscs, the 12-times PL enhancement was achieved in the hybrids of nanodisc arrays and monolayer MoS_2_ [19]. Among the various species of nanostructures, nanogaps have exhibited a prominent PL enhancement due to the EM field and the strong LSPR effect in the nanogap zone. The EM field intensity is strengthened as the nanogap size decreases, especially when the width of nanogaps is smaller than 10 nm [20,21]. Wang et al. reported a giant PL enhancement up to 20,000-fold for the hybrid of WSe_2_ on 12-nm nanotrenches [22]. In 2018, Cai et al. proposed a hybrid of MoSe_2_ on large area ultranarrow annular nanogap arrays (ANAs), which were fabricated using atomic layer deposition and polystyrene spheres lithography techniques [23]. Nanofabrication techniques, such as E-beam lithography (EBL) and focused ion beam (FIB) milling have been explored to directly fabricate 10-nm nanogap arrays [24,25,26]. However, the fabrication processes based on these techniques are relatively complex, due to fact that the small size of the nanogap is close to the limitation of resolution. In 2018, Hao et al. proposed a hybrid of MoS_2_ and patterned plasmonic dimers fabricated by a facile approach utilizing porous anodic aluminium oxide (AAO) templates during the angle-resolved shadow deposition [27]. The shadow deposition method, which is based on the inclined deposition of materials on the prefabricated pattern, has been demonstrated as a feasible way to fabricate 10-nm nanogap arrays over a large area [28,29]. However, the MoS_2_ hybrids based on 10-nm nanogap arrays fabricated by the shadow deposition method is still rarely reported.

In this work, a type of plasmonic hybrid composed of 10-nm Au nanogap arrays and monolayer MoS_2_ was proposed for PL enhancement. The 10-nm Au nanogap arrays were fabricated using the shadow deposition method, which was composed by depositing nanostrips with a 20 degree inclining angle on the nanostrips previously fabricated. By adjusting the length of nanogaps, the PL enhancement can be significantly boosted up to ~20-fold for the MoS_2_/nanogaps hybrid formed with 240-nm-length nanogaps under the excitation of a 532-nm laser. Combined with the finite-different time-domain (FDTD) simulation, the mechanism behind the PL enhancement was analyzed. Our results provide a feasible method to prepare large area MoS_2_-nanostructure plasmonic hybrids with a giant photoluminescence enhancement, promising their further applications in photodetectors, sensors, and emitters.

## 2. Materials and Methods

**Figure 1:**
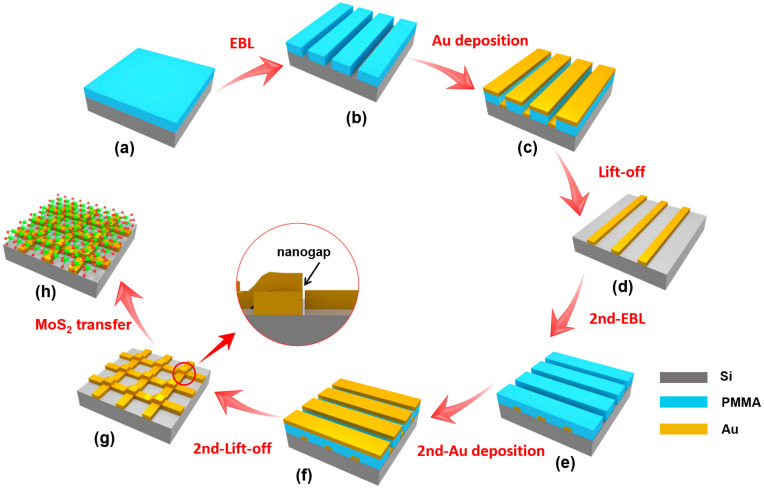
Schematic fabrication procedures of the MoS_2_/Au nanogap hybrid. (**a**) Substrate coated with polymethyl methacrylate (PMMA). (**b**) Nanotranches formed after the first E-beam lithography (EBL) process. (**c**) First Au deposition process. (**d**) Au nanostrips obtained after lift-off process. (**e**) Nanotrenches formed after the second EBL process. (**f**) The second Au shadow deposition process. (**g**) Nanogaps obtained by crossover of nanostrips. (**h**) Hybrid structure formed after MoS_2_ transferred. An enlarged view of the nanogap obtained was illustrated in the middle.

The monolayer MoS_2_ used in this work was fabricated on a single-crystalline sapphire substrate using the chemical vapor deposition (CVD) method [30]. The 10-nm nanogap arrays were fabricated using the shadow deposition method, a combination of EBL and electron beam deposition (EBD) techniques. Figure 1 illustrates the fabrication processes for the hybrid of MoS_2_ and nanogap arrays, which mainly include six steps. First, as presented in Figure 1a, a polymethyl methacrylate (PMMA) 495 A5 layer with a 200-nm thickness was spin coated onto the Si substrate, and the film was pre-baked on a hot plate at 180 °C for 60 s. Then, the EBL was used to write the patterns of nanostrips along the *y*-axis. Third, metallic nanostrips can be transferred by EBD on a 60-nm Au film and followed lift-off processes. After that, the nanostrips with a 60-nm-height were observed, as presented in Figure 1d. Fourth, the nanostrips along the *x*-axis were written by the alignment lithography, as illustrated in Figure 1e. Then, the shadow deposition of Au with a 20 degree inclining angle was applied to the Au strips formed in step 3, the thickness of Au was also 60-nm in the shadow deposition process, as shown in Figure 1f. Since the shadow origins from the deposition angle and steps, 10-nm nanogaps can be obtained, as illustrated in Figure 1g. Finally, the MoS_2_ was transferred on top of the nanogap arrays, forming a plasmonic MoS_2_/nanogaps hybrid, as shown in Figure 1h. The MoS_2_ flake was removed from the substrate to Au nanogap arrays using a PMMA-assisted wet transfer procedure, which has been applied to transfer 2D materials [22,31]. In order to investigate the plasmonic enhancement effect of the Au nanogaps on the PL emission of MoS_2_, another type of MoS_2_-nanogaps hybrid, in which the nanogap arrays were fabricated directly on the CVD-grown MoS_2_ on the sapphire substrate (nanogaps/MoS_2_) was fabricated for comparison.

**Figure 2:**
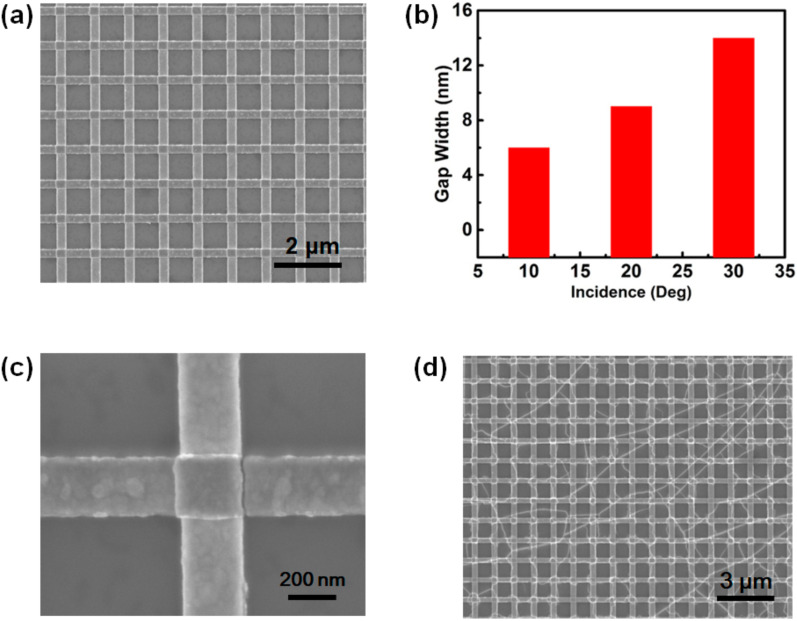
(**a**) scanning electron microscope (SEM) image of 10-nm nanogap arrays fabricated using the shadow deposition method. (**b**) Gap width obtained as a function of the inclining angle. (**c**) Magnified SEM image of a 10-nm nanogap of 240-nm-length. (**d**) SEM image for the nanogap arrays covered with MoS_2_.

The morphology and dimensions of the fabricated Au nanogaps are detected using a scanning electron microscope (SEM). Figure 2a presents the SEM images of the fabricated 10-nm Au nanogap arrays with a periodicity of 1 μm. It can clearly be seen that the nanogaps formed on the right side on each cubic island. Since the nanogaps were formed as a result of the shadow of first-layer nanostrips, the width of the nanogap obviously depended on the inclining angle for the deposition of the second-layer nanostrips. As exhibited in Figure 2b, the gap width widens with increasing the inclined deposition angle. The 10-nm nanogaps have been successfully fabricated by fixing the inclining angle at 20 degrees, as shown in Figure 2c. The length of this nanogap was determined as ~240 nm, since the line-width of the nanostrips was set at 240 nm. Therefore, the length of the nanogaps can be feasibly modulated by adjusting their line-width. Figure 2d shows the SEM image of the monolayer MoS_2_ transferred onto the nanogap arrays. It can be seen that the nanogap arrays are fully covered with the MoS_2_ monolayer.

**Figure 3:**
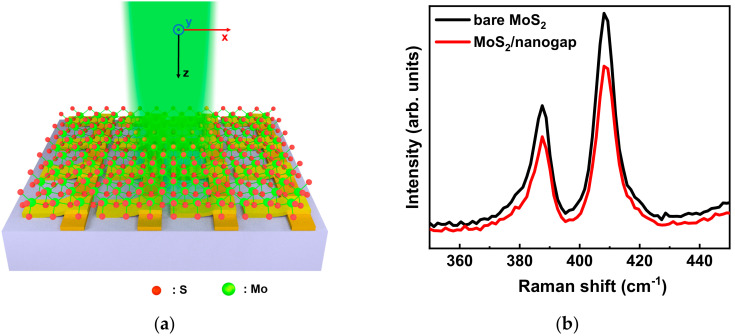

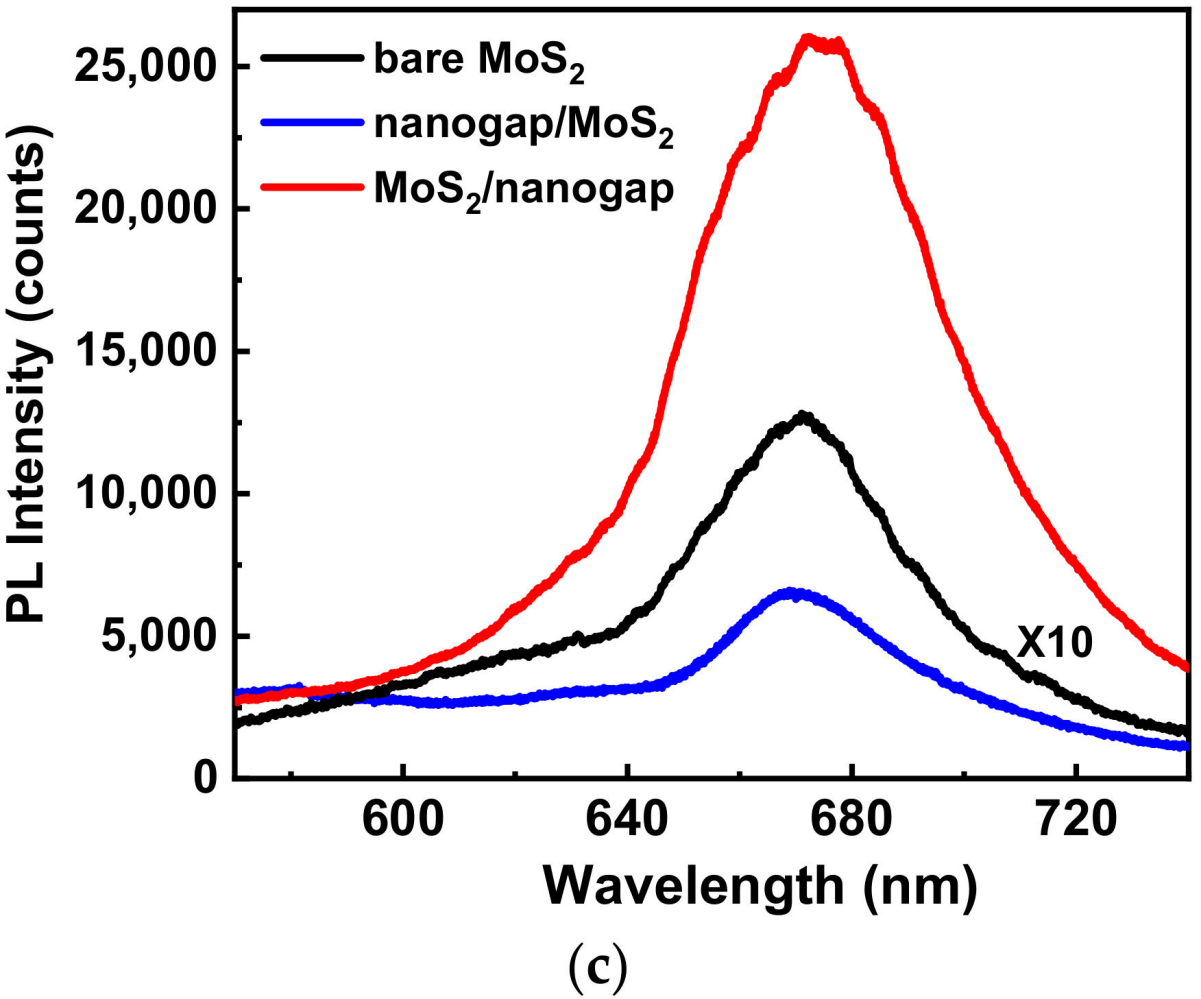
(**a**) Schematic illustration of the photoluminescence (PL) measurements on MoS_2_/nanogap hybrids. (**b**) Raman spectra for the bare MoS_2_ and MoS_2_/nanogap hybrid, respectively. (**c**) Typical PL spectra for the MoS_2_/nanogap hybrid, nanogap/MoS_2_ hybrid, and bare MoS_2_, respectively, collected under a 532-nm excitation.

Photoluminescence (PL) and Raman measurements were carried on a micro-confocal Raman spectrometer (Horiba HR Evolution) equipped with a microscope (BX41, Olympus, Tokyo, Japan). A 100× (NA = 0.9) objective lens was used for focusing the laser on the sample surface and collecting the PL signal. The 532-nm laser was used as the excitation sources for PL measurements and the laser power on the sample was about 0.5 mW in order to prevent the overheating effect. The spectra acquisition time was 30 and 10 s for Raman and PL acquisition, respectively. Figure 3a exhibits the schematic setup of the PL measurements on the MoS_2_/nanogap hybrids. As illustrated in Figure 3a, the laser beam was a normal incident on the sample surface, and the polarization direction of the incident laser was along the *x*-axis. In other words, the PL signal was collected while the incident polarization was perpendicular to the direction of the nanogap.

In order to solve the field enhancement effect of the nanogaps, the finite-different time-domain (FDTD) method, which was a state-of-the-art method for solving Maxwell’s equations in complex geometries, had been widely used in the nanogap configurations to collect the near-field distribution, transmission/reflection [23,32]. Here, in this work, the FDTD method was employed to simulate the electric field distribution on the 10-nm nanogap arrays. The geometries of the Au nanogaps in the simulations were designed to match the SEM images shown in Figure 2. The nanogaps chosen for the simulations were combined with steps with a 60-nm-height and the gaps that were built up by the shadow deposition with the width of the gaps were set to 10 nm. In addition, the gap length was determined by the linewidth of the nanostrip, which was used in the shadow deposition, and nanogaps with different lengths were simulated. The wavelength of the incidence light was set at 532 nm and propagated along the *z*-axis with the electric field polarized along the x- and y-axes, respectively. The source was a 1 × 1 μm Gaussian wave, which was similar to the excited laser used in the experiment. All the simulated boundaries were 12 layers perfectly matched layers (PML) to avoid reflections, and all the mesh steps along the x, y, and z-axes are set to 2 nm in order to obtain accurate results of the filed distribution in the nanogaps. Two monitors were placed perpendicular to capture the field distribution, one of them was perpendicular to the z-axes and overlapped the top surface of the structure, while the other was perpendicular to the y-axes and was placed in the nanogap. The filed distributions of the gaps with different lengths were collected.

## 3. Results and Discussion

Figure 3b presents the Raman spectra collected from a bare MoS_2_ and a hybrid MoS_2_/nanogap, respectively, which resembles the typical features of MoS_2_. It is worth noting that there is no obvious difference, such as frequency shift and peak broadening, between these two spectra suggesting that the hybridization with Au nanogaps did not introduce additional local strains or defects in the top MoS_2_ layer [33]. Figure 3c presents the typical PL spectra from two types of MoS_2_-nanogap hybrids (MoS_2_/nanogap and nanogap/MoS_2_) excited under a 532 nm laser. The length of the selected nanogap in the hybrids was 240 nm. The line shape of the PL spectra for the MoS_2_-nanogap hybrids are almost the same as that for the bare MoS_2_ (black curve), composed with an intense peak around 670 nm and a weak shoulder peak around 620 nm, which is consistent with those reported in the literature [34,35,36]. These two PL peaks are attributed as the A and B excitons, respectively, corresponding to the direct gap transition at the K point. Their energy difference is due to the spin-orbital splitting of the valence band. The A exciton for the bare MoS_2_ was centered at 670 nm, whereas the A exciton for the MoS_2_/nanogap hybrid red-shifts at 675 nm. One can see that the PL intensity from these two plasmonic hybrids displayed an obvious enhancement compared with the bare MoS_2_. Remarkably, the MoS_2_/nanogap hybrid exhibited an extraordinary PL enhancement with an amplitude up to 20-fold compared with the bare MoS_2_. In contrast, the PL intensity was just enhanced by five times while fabricating nanogaps arrays on top of MoS_2_. These results demonstrate that the MoS_2_/nanogap hybrid possesses a much better PL enhancement effect than the nanogap/MoS_2_ hybrid.

**Figure 4:**
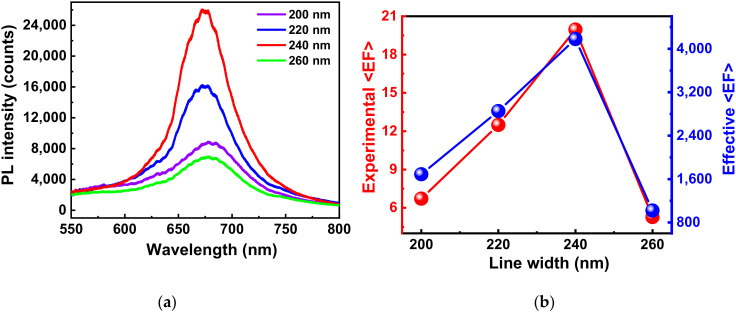
(**a**) Enhanced PL spectra for the MoS_2_/nanogap hybrids constituted of nanogaps with different lengths. (**b**) Experimental and effective PL enhancement factors as a function of the nanogap length.

Figure 4a presents the enhanced PL spectra for the MoS_2_/nanogap hybrids with lengths of 200, 220, 240, and 260 nm, respectively. It is noteworthy that the PL intensity of the MoS_2_/nanogap hybrids boosted with the increased line width of the nanostrips, and achieved the highest value for the 240-nm nanogap length. Then, the intensity of PL decreased quickly while the nanogap length increased to 260 nm. Using the PL intensity of the bare MoS_2_ as the reference, the PL enhancement of the hybrids was calculated as a function of the nanogap length. As shown in Figure 4b, the PL emission from the hybrids was increased from 7 to ~20 times, while the nanogap length increased from 200 to 240 nm, compared with the bare MoS_2_. With the increased nanogap length, the PL enhancement falls to 5-folds. The results in Figure 4 clearly demonstrate that the PL intensity enhancement is closely associated with the length of the nanogaps.

**Figure 5:**
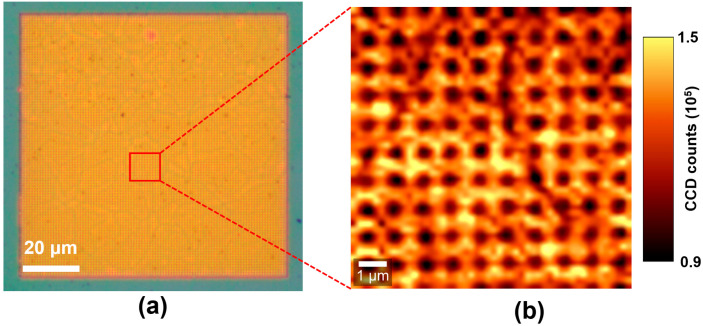
(**a**) Optical microscope image and (**b**) PL mapping image for the hybrid composed of the monolayer MoS_2_ and 240-nm-length nanogap arrays.

To see the intensity enhancement distribution on the plasmonic hybrid nanostructure, the PL mapping measurement was performed on a 10 × 10 μm^2^ area selected from the hybrid composed of the monolayer MoS_2_ and 240-nm-length nanogap arrays, as shown in Figure 5a. The mapping image was created by integrating the PL intensities over the range of 630–720 nm. As presented in Figure 5b, a gridding-like image for the PL distribution is observed, implying that the highest PL intensities appear at the intersections. The periodicity for meshes is 1 μm, which is consistent with that for the nanogaps fabricated in this work. This demonstrates that the highest PL intensities take place on the crossover of first- and second-layer nanostrips. This is attributed to the hot spots formed on the vertices of nanogaps due to the LSPR effect excited under a 532-nm laser. However, one cannot identify where the hot spots are specifically located in the nanogap, as the diameter of the laser spot is approximately 800 nm, which is much larger than the width (10 nm) and length (240 nm) of the nanogap. For future investigating the distribution of the hot spot in nanogaps, the probing technique with a higher spatial resolution, such as tip-enhanced Raman spectroscopy (TERS), is needed. The results in Figure 5 demonstrate that the nanogap arrays fabricated in this work were able to enhance the PL emission of MoS_2_ prominently. Moreover, the nanogap arrays were fully covered by the monolayer MoS_2_. The differences in the enhanced PL intensity between the nanogaps could be attributed to the defects or strains generated during the MoS_2_ transferring procedure.

**Figure 6:**
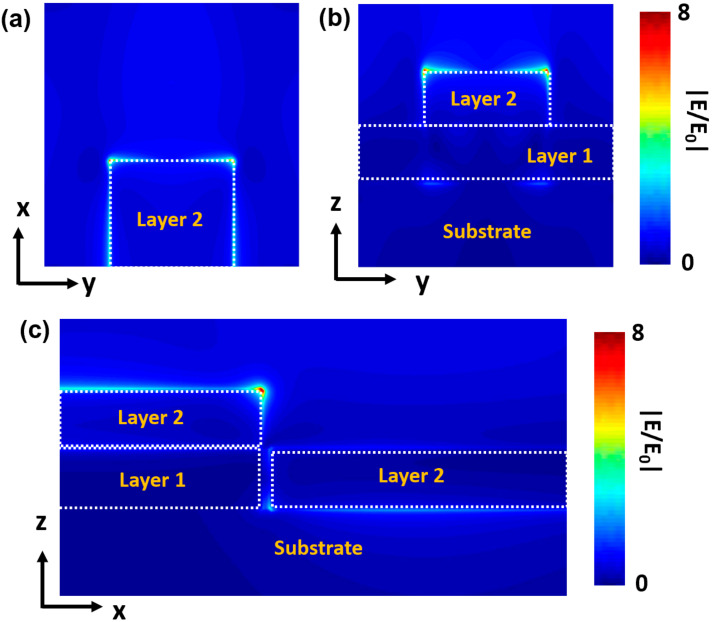
(**a**) Top view and cross-sectional view for the (**b**) z-y plane and (**c**) x-z plane of the calculated electric field distribution on the 10-nm Au nanogap with 240-nm-length excited by a 532-nm laser.

In order to understand the physical mechanism behind the PL enhancement for the MoS_2_/nanogap hybrid, the FDTD simulation was employed to evaluate the electromagnetic field distribution of the 10-nm Au nanogap under a 532-nm excitation. Figure 6 shows the map of the simulated electric field enhancement |E/E_0_| on the surface of the nanogap formed of 240-nm-width nanostrips under x-polarization. E is the local electric field at the surface and E_0_ is the incident electric field. One can see in Figure 6a that the maximum enhancement of the electric field takes place at the two vertices of the second layer nanostrip, which is ~8 times under the 532-nm excitation. In contrast, the electric field at the interface between the first layer nanostrip and substrate does not exhibit an obvious enhancement, as exhibited in Figure 6b. Figure 6c shows the field distribution in the x-z plane, in which one can see that the hot spots do not exhibit the same amplitude on the two sites of the nanogap. A bright hot spot emerges at the vertices of the second layer on the left side, while a weaker spot appears at the vertices of the second layer on the right side. This may be attributed to the height difference of 60 nm between the two sides of the nanogap, so that the plasmon coupling in the nanogap zone is not prominent. As demonstrated in previous literatures, the literal dimension of the nanogap is normally very small, for example, nanoparticle dimer or nanotips, so that the EM field can be confined in a limited space and significantly enhanced. The interaction of plasmons along the interparticle axis of a nanoparticle dimer results in low-energy longitudinal bonding dipole plasmon (LBDP) modes [37,38]. However, the length of the nanogap in our work is too long, the surface plasmon can propagate along the edges of the nanogap. The EM field cannot uniformly be distributed on the whole nanogap zone, and the highest EM field (hot spots) only takes place on the vertices of the nanogaps. Therefore, the hot spots on the 240-nm-length nanogap primarily arise from the LSPR rather than the plasmon coupling modes.

The results in Figure 6 clearly explain why the PL intensity for the MoS_2_/nanogap hybrid is much higher than that for the nanogap/MoS_2_ hybrid (see Figure 3c). The discrepancy in the PL enhancement between these two types of hybrid structures could be attributed to their own architectures, in other words, the location of the monolayer MoS_2_ in the hybrids. As it has been demonstrated, the EM field enhancement is a type of near field effect, which requires the molecule to be within a few nanometers from the surface of the plasmonic nanostructure for an appreciable enhancement in order to be obtained [39,40]. For the MoS_2_/nanogap hybrid structure, the monolayer MoS_2_ is transferred on top of the nanogap. The hot spots excited by the 532-nm laser can directly interact with MoS_2_, consequently enhancing the PL emission of MoS_2_. In sharp contrast, MoS_2_ is under the nanogap structures in the nanogap/MoS_2_ hybrid structure, which is 60-nm far away from the hot spots, the LSPR cannot interact with the MoS_2_ layer, so the PL intensity of MoS_2_ cannot be enhanced obviously.

**Figure 7:**
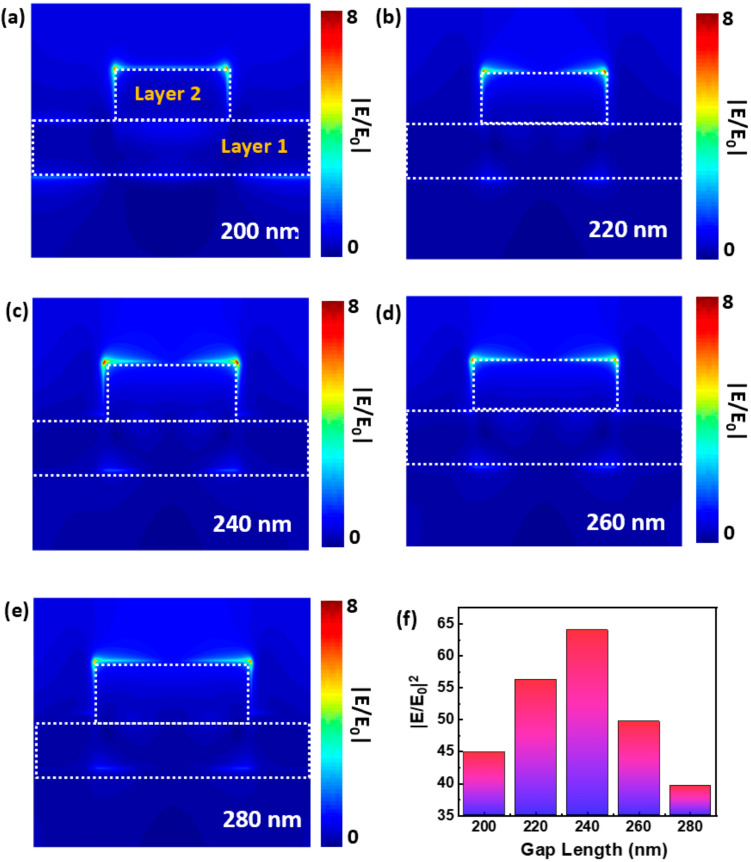
(**a**–**e**) Cross-sectional view of the simulated electric field distribution on the 10-nm Au nanogaps with selected gap lengths. (**f**) Calculated electric field enhancement of the 10-nm Au nanogap as a function of gap length.

Figure 7 presents the cross-sectional view of the simulated electric field enhancement |E/E_0_|on the nanogaps with a length of 200, 220, 240, 260, and 280 nm, respectively. One can see that the maximum electric field enhancement (“hot spots”) appears at the top two corners of the second layer for all the nanogaps. Noteworthily, the maximum electromagnetic field enhancement takes place at the nanogap with a 240-nm-length. However, the difference of the field enhancement between these nanogaps is not obvious. Therefore, the enhancement of the light at the hot spots was calculated using |E/E_0_|^2^ and presented in Figure 7f. As illustrated clearly in Figure 7f, the light (near field) was enhanced by ~64-fold for the nanogap with a 240-nm-length, whereas the field EF is down to 40-fold for the 280-nm nanogap length.

The simulation results in Figure 7f imply the significant light enhancement ability of these nanogap nanostructures. However, the maximum measured PL enhancement for the MoS_2_/nanogap hybrids is just 20 times (see Figure 4b), which is lower than the simulated results. This phenomenon could be attributed to the architecture of the hybrids, in which only the nanogap area has contributions to the PL enhancement of MoS_2_, as exhibited in Figure 7. In order to evaluate the PL enhancement of these nanogaps, the PL enhancement factor was corrected using the area of nanogaps. We calculated the effective average PL enhancement factor, 〈EF〉, using the following formula [22,41,42]:

(1) $$\langle \mathrm{EF}\rangle =\frac{I_{hybrid}}{I_{bare}}\frac{A_{bare}}{A_{hybrid}}$$

where *I_hybrid_* is the PL intensity from the MoS_2_/nanogap hybrids and *I_bare_* is the PL intensity from the bare MoS_2_. *A_bare_* represents the excitation area of the laser spot size (π × 400^2^ nm^2^) and *A_hybrid_* represents the area of the nanogaps (10 × gap length nm^2^) within the laser spot, which depends on the length of nanogaps. The effective 〈EF〉 were calculated using Equation (1) and plotted as a function of the length of nanogaps in Figure 4b. Remarkably, the estimated 〈EF〉 reaches a factor up to ~4180 for the hybrid with 240-nm-length nanogap. The maximum effective EF is much higher than that of the simulated EM EF (~64). This is due to the fact that the effective PL enhancement is actually composed of both the plasmon-enhanced excitation process and the plasmon-enhanced emission process [22]. The simulation results in Figure 7f just present the EF for the excitation process. If the EF for the plasmon-enhanced emission is taken into consideration, the simulated EF would be at the same level of the effective EF.

Moreover, the effective 〈EF〉 decreased with the increasing gap length, and down to 210 for the 280-nm-length nanogap, which follows the similar trace as the experimental 〈EF〉, as shown in Figure 4b. The huge discrepancy between the experimental and effective 〈EF〉 should be attributed to the small effective interaction area between the nanogap and MoS_2_. As shown in Figure 6, only the top corners of the nanogap exhibit a high electromagnetic field enhancement, whereas the other part of the nanostrips does not make dominating contributions to the PL enhancement. In order to easily compare the PL enhancement for the hybrids, the periodicity of the nanogap arrays is the same (1 μm) for all the samples used in this work. In other words, there is only 1 nanogap within the laser spot. Therefore, in the future device fabrication process, increasing the density of nanogaps would be a proper route to enhance the PL emission of MoS_2_.

## 4. Conclusions

In summary, we have proposed a type of plasmonic hybrid for enhancing the PL emission of MoS_2_ by fabricating 10-nm-wide Au nanogap arrays on the monolayer MoS_2_. By taking advantage of the LSPR arising in the nanogaps, the PL emission of MoS_2_ was significantly enhanced under a 532-nm excitation, which can be modulated by adjusting the width of the nanostrips. The maximum PL enhancement was demonstrated on the MoS_2_/nanogaps hybrid formed with nanogaps of 240-nm-length, in which an effective emission enhancement of ~20-fold is attained. The effective enhancement factor for the MoS_2_/nanogaps hybrid has achieved ~4180. The mechanism for the PL enhancement of the MoS_2_/nanogaps hybrids was studied using the FDTD simulation. Our results demonstrate the feasibility of a giant photoluminescence enhancement for this hybrid of MoS_2_/10-nm nanogaps, promising their further applications in photodetectors, sensors, and emitters.
